# A Dynamic Jaw Model With a Finite-Element Temporomandibular Joint

**DOI:** 10.3389/fphys.2019.01156

**Published:** 2019-09-13

**Authors:** Benedikt Sagl, Martina Schmid-Schwap, Eva Piehslinger, Michael Kundi, Ian Stavness

**Affiliations:** ^1^Department of Prosthodontics, University Clinic of Dentistry, Medical University of Vienna, Vienna, Austria; ^2^Institute of Environmental Health, Medical University of Vienna, Vienna, Austria; ^3^Department of Computer Science, University of Saskatchewan, Saskatoon, SK, Canada

**Keywords:** jaw modeling, temporomandibular joint, finite-element modeling, musculoskeletal modeling, computational biomechanics

## Abstract

The masticatory region is an important human motion system that is essential for basic human tasks like mastication, speech or swallowing. An association between temporomandibular disorders (TMDs) and high temporomandibular joint (TMJ) stress has been suggested, but *in vivo* joint force measurements are not feasible to directly test this assumption. Consequently, biomechanical computer simulation remains as one of a few means to investigate this complex system. To thoroughly examine orofacial biomechanics, we developed a novel, dynamic computer model of the masticatory system. The model combines a muscle driven rigid body model of the jaw region with a detailed finite element model (FEM) disk and elastic foundation (EF) articular cartilage. The model is validated using high-resolution MRI data for protrusion and opening that were collected from the same volunteer. Joint stresses for a clenching task as well as protrusive and opening movements are computed. Simulations resulted in mandibular positions as well as disk positions and shapes that agree well with the MRI data. The model computes reasonable disk stress patterns for dynamic tasks. Moreover, to the best of our knowledge this model presents the first ever contact model using a combination of EF layers and a FEM body, which results in a clear decrease in computation time. In conclusion, the presented model is a valuable tool for the investigation of the human TMJ and can potentially help in the future to increase the understanding of the masticatory system and the relationship between TMD and joint stress and to highlight potential therapeutic approaches for the restoration of orofacial function.

## Introduction

The masticatory system is an incredibly complex musculoskeletal system, comprised of two bony structures, the mandible and the skull, which are connected by two temporomandibular joints (TMJs). The masticatory system is frequently used in everyday tasks like speaking and chewing, but a variety of complications can lead to dysfunction of the TMJs. Disorders of the TMJ are grouped as temporomandibular disorders (TMDs) (Schiffman et al., 2014) and are prevalent in roughly 20% of the population (Solberg et al., 1979). Problems range from reduced quality of live up to severe impairment of the above-mentioned essential functions.

One unique feature of the masticatory system is that it encompasses two separate joints that articulate the mandible and hence allow a multitude of different movement patterns with six degrees of freedom including rotation as well as translation (Drake et al., 2014). Moreover, a dynamic, cartilaginous disk is located between the mandibular condyle and the articular fossa. This TMJ disk plays a crucial role in force absorption and lubrication of the joint (Koolstra et al., 2007; Tanaka and Koolstra, 2008; Koolstra and Tanaka, 2009; Nickel et al., 2009; Stankovic et al., 2013) and additionally increases the complexity of mandibular dynamics. Previous literature suggests that many cases of TMD are associated with increased load on the TMJ (Detamore and Athanasiou, 2003; Ingawale and Goswami, 2009), which highlights the importance of the detailed investigation of TMJ loads using realistic, dynamic loading scenarios. Additionally, the small size and complex organization of the TMJ make *in vivo* investigations of joint forces impossible due to patient safety restrictions. This leaves computational biomechanics as one of the few possibilities to gain knowledge on the internal workings of the TMJ.

Historically speaking, computational models of the masticatory region can be separated into two categories. On the one hand, there are rigid body models which have been extensively used for most of the history of masticatory computational modeling (Throckmorton and Throckmorton, 1985; Koolstra and van Eijden, 1995; Trainor et al., 1995; Peck et al., 2000). These dynamic models are a valuable tool for the investigation of complex movements or muscle activation patterns. Rigid body models have been used previously to investigate a variety of tasks, including open-close movements (Tuijt et al., 2010) and mastication (Hannam et al., 2008). Moreover, they have been used to investigate changes of function due to surgery (de Zee et al., 2009; Stavness et al., 2010) or the effect of morphological changes on the involved structures (Marková and Gallo, 2016). Their main drawback is that they use quite simple representations of the TMJ itself, consisting of a combination of planes or spline functions and completely lacking a TMJ disk (Curtis, 2011; Hannam, 2011). Hence, these models cannot appropriately capture the forces applied to the different joint structures.

The second category of jaw simulations are finite element (FE) models. FE models of the masticatory region generally are comprised of detailed meshes of the structures of the TMJ and enable the use of sophisticated material models (Lamela et al., 2011; Commisso et al., 2016). While this set up allows researchers to compute more realistic force patterns of the joint, simulations often solely focus on the TMJ itself; neglecting or drastically simplifying the dentition as well as muscle force calculations. Additionally, due to the high computational cost of these models, typically static- or quasi-static simulations are performed (Mori et al., 2010; Commisso et al., 2014; Hattori-Hara et al., 2014). However, tasks that are most likely to create high forces in the joint, like mastication or tooth grinding, have important dynamic characteristics and hence cannot be appropriately modeled using a static or quasi-static set-up. This problem becomes more apparent when investigating the wide range of functional and parafunctional movements, which are performed many times during the course of a single day and involve large translations and rotations.

Overall, the above-mentioned facts highlight the need for representations of dynamic jaw movement and muscle forces, while also having a detailed representation of TMJ and disk mechanics. Such a model enables the dynamic investigation of functional or non-functional movements; giving detailed joint stress or strain predictions that are transferable to the *in vivo* situation. To the best of our knowledge only two models exist that fit this description. The model proposed by Koolstra and van Eijden is the only previous attempt of a dynamic musculoskeletal model of the full masticatory system with a FE model of the TMJ (Koolstra and van Eijden, 2005). Nonetheless, the model includes a quite coarse joint representation, most likely due to the computational limitations at the time of publication. Moreover, the model was created for the right side of the TMJ and mirrored. This neglects the effect of facial asymmetries, which are very common in humans. The second model developed by Martinez Choy et al. (2017) proposes a full FEM modeling approach, containing meshes of all involved structures as well as Hill-type muscles. The model was built using data from various literature sources and the anatomy was based on the (Koolstra and van Eijden, 2005) model, using a mirrored jaw setup. The model was used for the investigation of a chewing cycle. A similar modeling approach has been previously used for the evaluation of mastication in mice (Tsouknidas et al., 2017). Due to the full FEM approach a long simulation time on even the most powerful PCs is to be expected, even though no simulation time was reported. These long simulation times limit the applicability of the model in a clinical setting.

The aim of this project was to create a comprehensive rigid body model, derived from medical imaging data, encompassing the whole masticatory region combined with a detailed representation of the TMJ, while keeping simulation times reasonably short. To validate the model, we computed opening and protrusion movements and compared mandibular end position as well as disk position and deformation to high-resolution MRI data from the same volunteer. Moreover, we report clenching stresses to more thoroughly investigate the behavior of the model. Simulation times are also reported and compared to previous models. Overall, this project presents a unique tool for the investigation of the workings of the jaw region that is well-suited for future analysis of the effect of various masticatory functions, parafunctions and dysfunctions and consequently could give valuable input for the development of novel treatment strategies of orofacial sensorimotor impairments.

### Related Work

#### Rigid Body Modeling of the TMJ

Early computational investigations of the masticatory region were mostly performed using two-dimensional rigid body models. These investigations focused on static investigations of joint reaction forces utilizing muscle force estimations derived from maximum bite force estimations (Greaves, 1978; Throckmorton and Throckmorton, 1985). While these investigations are a valuable tool for the examination of bite performance and the mechanical efficiency of masticatory muscles, they cannot be used for dynamic investigations and are an oversimplification of the three-dimensional masticatory system. An early example of a three-dimensional dynamic model was published by Koolstra and van Eijden (1997). The TMJ was modeled as purely elastic, frictionless contact between a sphere (simplified condyle) and a spline surface representing the articular fossa. Their model used contact points on the lower teeth and a flat surface mimicking the occlusal surface of the upper dentition. Moreover, the presented model includes a muscle model that connects muscle force to muscle activation, muscle length and a force-velocity curve. Tooth contact was modeled as contact between points representing the tip of the lower teeth and a plane that represented the occlusal plane. In a more recent iteration of the model (Tuijt et al., 2010) the TMJ surfaces were modeled as 3D shell type meshes. An ellipsoid was used for the condyle and the fossa was modeled using a third-degree polynomial in the sagittal plane, combined with a second-degree polynomial for the mediolateral curve. In this model a tangent plane approximation of the fossa mesh around a contact point was used. Penetrating vertices were defined, and point-to-plane distance was calculated to derive the joint reaction force for each penetrating vertex. The upper teeth were modeled as single bite plane and the lower dentition was modeled using points for an incisor and the two second molars.

Peck et al. (2000) also used an ellipsoidal shape to approximate the condyle and a combination of multiple linear plates to model the condylar path along the fossa. Again, contact was monitored by interpenetration of the two geometrical shapes. In the case of constant contact an instantaneous constraint was added to simulate sliding along the surfaces. Dentition was simulated as a flat occlusal plane and muscles were modeled as Hill-type actuators (Hill, 1953). The Stavness et al. (2006) model can be seen as most recent version of this “model family.” The model uses a bilateral or unilateral point constraint, sitting in the anatomical center of the condyle and a combination of three rigid, frictionless surfaces. These surfaces define the movement of the mandible in the anterior–posterior and medial-lateral directions. de Zee et al. (2007) used a comparable approach modeling the TMJ by using a single unilateral, planar constraint that was angled downwards and canted medially. In a more recent version, the group used an elastic contact foundation model to solve contact between the condyle and the fossa articularis using a Force Dependent Kinematics approach to track movement data and compute muscle, ligament and contact forces (Andersen et al., 2017).

#### Finite Element Modeling of the TMJ

Nagahara et al. (1999) investigated the stress distribution and displacement during static clenching. This early investigation used a CT scan of a cadaver for bony structures and the TMJ disk was digitized after extraction. Muscle forces were modeled using external forces in the direction of the main closing muscles. Perez del Palomar and Doblaré created FEM simulations of mouth opening as well as lateral movements (Pérez del Palomar and Doblaré, 2006a, b). The models were built from medical scans of a patient and used a porohyperelastic material model for the TMJ disk. No muscle representations were included, and the movements were simulated by prescribed translation of the mandible. While the model used for opening simulations only contains one half of the masticatory system, the model for lateral movements contains both joints. Mori et al. (2010) built a model from 1.5T MRI images of a volunteer. The cartilaginous and ligamentous structures were modeled using a Kelvin material model and retrodiscal tissue and the TMJ capsule were included. Moreover, the articular cartilage was included as uniform layer. Mandible movement was constrained to only allow movement in the sagittal plane and clenching was simulated using an external load. Hattori-Hara et al. (2014) created a model of the TMJ from a CT scan and a 1.5T MRI of a patient for the investigation of unilateral disk displacement during static clenching. The model includes the bony structures and TMJ disk. Articular cartilage and capsule were modeled as uniform layers. Muscle forces were modeled as external forces and distributed over the insertion of the muscles. Commisso et al. (2014) created a model of the mandible and TMJ from a human cadaver. The model includes cube like teeth that are connected to the mandible with a layer of elements mimicking the periodontal ligament. A quasi-linear viscoelastic material was used for the TMJ disk. Forces were applied as external load at the insertion area of each respective muscle. They used the model to simulate sustained clenching as well as rhythmic masticatory muscle activity. In a more recent study they used the same model to investigate the lateral pterygoid muscle during a unilateral mastication cycle (Commisso et al., 2015). For this purpose, they used a two-step setup. They estimated muscle forces using a Hill-type muscle model, while neglecting force-length as well as force-velocity dependencies. These calculated muscle forces were applied to the muscle insertion areas as external forces. Martinez Choy et al. (2017) proposed a full FEM modeling approach, containing detailed meshes of all involved structures as well as Hill-type muscles. The model was built using data from various literature sources and used for the investigation of a chewing cycle.

Recently, co-simulation techniques have been proposed to use musculoskeletal models to define boundary conditions for static FEM simulations. Examples of modeled joint systems include tibial loading while load carrying (Xu et al., 2019) or patellofemoral cartilage stresses during a stair climb task (Pal et al., 2019). Currently, no such approach has been reported for jaw models, even though the recent, more sophisticated rigid body models are theoretically capable of driving such a modeling strategy (Andersen et al., 2017). Nevertheless, this approach does not fully solve the presented problems. While using such a technique potentially decreases the simulation time needed, the use of two different modeling toolkits increases the complexity of model setup and therefore decreases the likelihood of clinical use. Moreover, the use of forces computed with a simple joint set-up might not necessarily compute the correct motion and reaction forces when applied to a more complex FEM joint. To the best of our knowledge the only previous dynamic rigid body model that incorporated a FEM TMJ was published by Koolstra and van Eijden (2005). The mandible was modeled as dynamic rigid body with 12 Hill-type actuators attached and the TMJ disks were included as FE models with tetrahedral elements and an edge length of approximately 0.5 mm. The articular cartilage was represented as a uniform layer using a FE approach. The model was built from cadaver data of the right TMJ and mirrored for the left side. A maximum jaw opening of 3 cm was achieved. Table 1 presents an overview of the literature review.

**TABLE 1:** Overview of features of computer models of the jaw region.

**References**	**Point constraint**	**Geometric contact**	**FEM disk**	**Deformable articular cartilage**	**FEM capsule/ligaments**	**Teeth can be in and leave contact**	**Dynamic**	**Muscle driven**	**Individually modeled joints**	**Reported simulation time**
Koolstra and van Eijden, 1997	×	✓	×	×	×	✓	✓	✓	×	×
Tuijt et al., 2010	×	✓	×	×	×	✓	✓	✓	×	×
Peck et al., 2000	×	✓	×	×	×	✓	✓	✓	×	×
Hannam et al., 2008	✓	×	×	×	×	✓	✓	✓	×	×
de Zee et al., 2007	✓	×	×	×	×	✓	✓	✓	×	×
Andersen et al., 2017	×	✓	×	×	×	✓	✓	✓	✓	×
Nagahara et al., 1999	×	×	✓	×	×	×	×	×	×	×
Pérez del Palomar and Doblaré, 2006a	×	×	✓	×	✓	×	×	×	×	×
Pérez del Palomar and Doblaré, 2006b	×	×	✓	×	✓	×	×	×	✓	×
Mori et al., 2010	×	×	✓	✓	✓	×	×	×	✓	×
Hattori-Hara et al., 2014	×	×	✓	✓	✓	×	×	×	✓	×
Commisso et al., 2014 + 15	×	×	✓	×	✓	✓	×	RB model for muscle forces	✓	×
Martinez Choy et al., 2017	×	×	✓	✓	✓	✓	✓	✓	×	×
Koolstra and van Eijden, 2005	×	×	✓	✓	×	✓	✓	✓	×	×
Our model	×	×	✓	✓	×	✓	✓	✓	✓	✓

## Materials and Methods

Data were acquired from one symptom-free volunteer. Ethics approval was obtained from the institutional review board of the Medical University of Vienna and written informed consent was obtained. The detailed data acquisition and processing workflow has been extensively explained in a previous publication (Sagl et al., 2019b). In brief, we collected a single full skull CT scan for bony structures as well as a full skull MRI scan for the definition of muscle paths. Additionally, high resolution TMJ MRI volumes were created to enable accurate representations of the TMJ disks. MRI scans were acquired using a TSE-T1 sequence on a Siemens Magneton 3T machine and a 64-channel head coil, achieving a resolution of 0.17 mm and a slice thickness of 1 mm. High-resolution scans were collected in different static positions, using silicone bite blocks for defined jaw postures verified with the help of a jaw tracking system. These scans were used for model validation as described below. Bony structures were segmented from the CT scan, while the TMJ disk was manually segmented from the MRI scans by an expert specialized in TMD and TMJ-MRI for all positions. To enable realistic maximum opening behavior with a static hyoid, the respective mesh was moved 7 mm posteriorly and 7 mm downwards, which lies in the range of previously reported literature values (Muto and Kanazawa, 1994).

The model was developed using the opensource ArtiSynth Modeling Toolkit^1^ (Lloyd et al., 2012). Boney structures were modeled as rigid bodies. Inertial properties of the mandible were estimated from mesh geometry with an assumed mandibular mass of 200 g (Langenbach and Hannam, 1999). The hyoid and skull were kept static for all presented simulations and therefore no inertia properties had to be defined.

**FIGURE 1:**
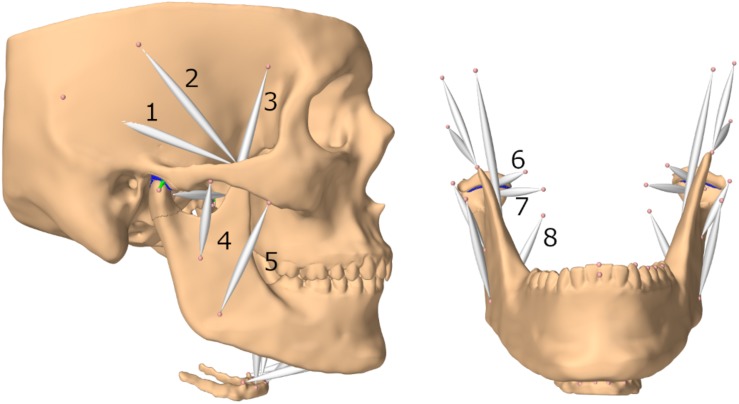
Lateral view of model and frontal view without skull (submental muscles not labeled); (1) posterior part of temporalis muscle (2) medial part of temporalis muscle (3) anterior part of temporalis muscle (4) deep masseter muscle (5) superficial masseter muscle (6) superior head of lateral pterygoid muscle (7) inferior head of lateral pterygoid muscle (8) medial pterygoid muscle.

Muscles were represented as Hill-type point-to-point muscles (Hill, 1953; Peck et al., 2000; Hannam et al., 2008). Since these muscle models apply forces in the one-dimensional direction of the force vectors defined by an origin and insertion point, larger muscles were split up into multiple models to more accurately mimic activation of muscle compartments. The included muscles are: posterior, medial and anterior parts of the temporal muscle, superior and inferior head of the lateral pterygoid muscle, superficial and deep masseter muscles, medial pterygoid muscle, anterior digastric muscle, geniohyoid muscle, anterior and posterior mylohyoid muscle (Figure 1). Cross-sectional areas, velocity-force and length-force behavior were defined with the help of previous literature (Langenbach and Hannam, 1999; Peck et al., 2000; Hannam et al., 2008).

The TMJ disks were modeled as FEM with roughly 6000 first-order tetrahedral elements for the right and 8000 for the left disk and an estimated weight of 0.006 kg. The volumetric meshes were created using the tetgen library and the previously segmented and processed disk surface meshes (Si, 2015). A hyperelastic Mooney–Rivlin material with material constants taken from literature (Beek et al., 2001; Koolstra and van Eijden, 2005) (*C*_1_ = 9⋅10^5^*Pa* and *C*_2_ = 9⋅10^2^*Pa*), was used. The strain energy function for this material is:

(1) $$W\,\left(I_{1},I_{2}\right)=C_{1}\,\left(I_{1}-3\right)+C_{2}\,\left(I_{2}-3\right),$$

where *C*_*1*_ and *C*_*2*_ are material constants and *I*_*1*_ and *I*_*2*_ are the first and the second invariant of the left Cauchy–Green deformation tensor *B*. Simulations were computed using a first-order Backward Euler implicit integrator.

**FIGURE 2:**
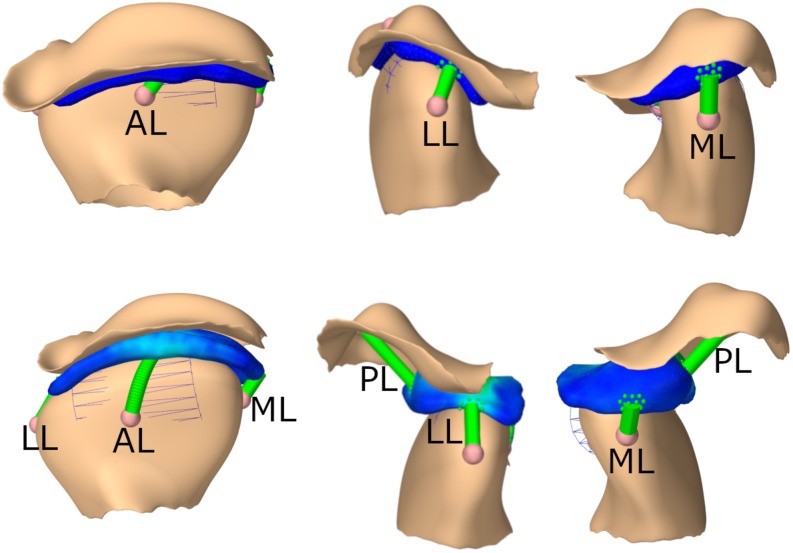
TMJ capsule representation from front, lateral, medial; First row: closed state, second row: opened position. AL, anterior ligament; LL, lateral ligament; ML, medial ligament; PL, posterior ligament.

Additionally, four axial springs mimic the biomechanics of the TMJ capsule. One connects the disk and the condyle anteriorly, one medially, one laterally and one connects the skull and disk posteriorly. The ligaments were modeled as inextensive cables, using an elongation stiffness of 250 MPa once the tendon slack length was exceeded. For the posterior ligaments the slack length was 7.5 mm longer than the initial length of the ligament, for the anterior ligaments the slack length was 4 mm longer and for the medial the slack length was 1.9 mm and for the lateral ligament 2.5 mm longer than the initial length. These slack lengths were determined to ensure best simulation results for various movements. A wrapping cylinder was added on both sides to make the anterior ligament wrap around the mandibular condyle. Since attaching the axial springs to a single node of the FEM would lead to unstable and incorrect simulation results, we used a distributed FEM attachment, spreading out the applied force over approximately 20 nodes per ligament; an approach similar to previous literature (Fernandez et al., 2014). An overview of the joint set-up can be found in Figure 2.

To speed up simulations, articular cartilage was modeled as an elastic foundation (EF) contact model (Blankevoort et al., 1991; Bei and Fregly, 2004), as opposed to full FEM for the contact surfaces. EF contact can be seen as an elastic layer bonded to a rigid substrate. This is achieved by distributing springs over the surface of the rigid body, modeling the elastic layer with a predefined thickness. EF contact approaches speed up contact calculation by neglecting the effect of contact forces applied at one location at all other locations. This simplification speeds up simulations substantially and has proven to be a valuable tool for elastic layers that are connected to a rigid body (Bei and Fregly, 2004). The EF layers had a thickness of 0.4 mm (Hansson et al., 1977) and contact pressure between the surface mesh of the temporomandibular disk (TD) and the articular cartilage mesh (AC) was computed using:

(2) $$p\,\left(d\right)=K\,\ln\left(1-\frac{d}{h}\right),K\equiv \frac{-\left(1-\nu \right)\,E}{\left(1+\nu \right)\,\left(1-2\,\nu \right)}$$

where *E* is the elastic modulus, ν is the Poisson’s ratio of the contact, *d* represents the depth of penetration and *h* is the cartilage thickness. Contact pressure is non-linear with respect to *d* and linear with respect to *E*. We based our elastic modulus on previous literature values for an articular cartilage Mooney–Rivlin material (Koolstra and van Eijden, 2005) and estimated *E* using 6⋅*C*_1_ (Nazari et al., 2011). For our simulations, we used an elastic modulus of 2.7 MPa and a Poisson’s ratio of 0.49. Contact between the TD and the AC is computed by finding the FEM nodes of the TD that penetrate the AC mesh. For each penetrating note a contact, with a penetration depth *d* and normal direction *n*, is defined. Equation (2) is then used to determine the appropriate nodal response force *f*, according to

f=p⁢(d)⁢A⁢n

Here *A* denotes the surface area associated with the contact (estimated by splitting the total mesh penetration area over all penetrating vertices).

Since usually contact forces are stiff, very small timesteps or the use of an implicit integrator are required to keep simulations stable using an EF approach. To additionally increase stability, our implementation uses a constraint regularization scheme that does not use penalty forces to simulate contact, but rather uses point constraints, based on the contact normal directions. A “contact force behavior,” which implements the EF approach, is then used to regularize, or “soften” the contact. Full details are given in Servin et al. (2006).

To validate the model, we performed multiple forward-dynamics simulation tasks. First, we computed a postural rest position. Additionally, an active maximum opening movement was performed (bilateral maximal activation of lateral pterygoid, anterior digastric, geniohyoid, and mylohyoid muscles) as well as an incisal edge-to-edge protrusion movement (bilateral maximal activation of lateral pterygoid muscles, with lower activation of jaw closing and opening muscles for stabilization). Maximal activation of the jaw closing muscles was used to simulate a clenching behavior. Simulated end positions of the mandible and TMJ disks for active opening and protrusion were compared to the respective meshes segmented from the high-resolution MRI images of the same volunteer. Additionally, differences in deformation of the TMJ disks were visualized using these MRI data. Moreover, to check the mesh quality, we computed the mean values for the ratio of the radius of the circumscribed sphere to the shortest edge length as well as for the maximal dihedral angle for both disks to measure quality of the created first-order tetrahedral elements.

As an additional verification of the mesh used in the model, the opening task was simulated with different numbers of mesh elements. For this purpose, the surface meshes of both disks were remeshed, using Meshmixer (Autodesk, Inc.), to have an edge length of 0.5/0.45/0.4/0.35/0.3 mm respectively. This led to disk models with numbers of elements varying from approximately 7500 to 20000 elements for the left disk. The overall trend in stress maps for the left disk was compared between the simulations to check for consistency. Furthermore, we performed a trend validation, describing TMJ disk stress and qualitatively comparing it to previous work.

To investigate the effect of the chosen material constants for the FEM disks, a sensitivity analysis consisting of 15 simulations was performed, including changes of both material constants over a range of one negative and positive order of magnitude for C1 and two negative and positive orders of magnitude for C2.

## Results

A postural rest position simulation, with a steady-state activity of 0.08% for the closer muscles (masseter, temporalis, and medial pterygoid muscles) (Langenbach and Hannam, 1999), produced a mouth with an inter-incisal separation of 5 mm. For maximum opening an inter-incisal gap of 30 mm was achieved.

**FIGURE 3:**
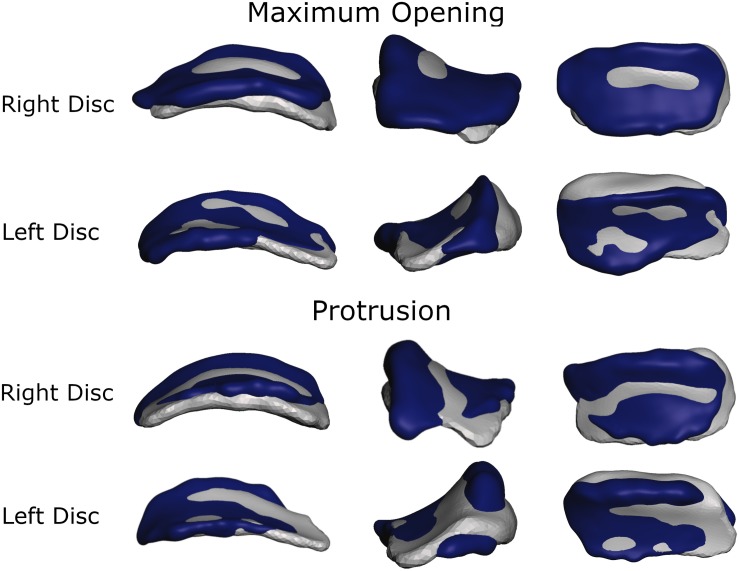
Overlay of simulation (gray) and MRI data (dark blue) for comparison of disk position and shape for maximum opening and edge-to-edge protrusion simulations.

**FIGURE 4:**
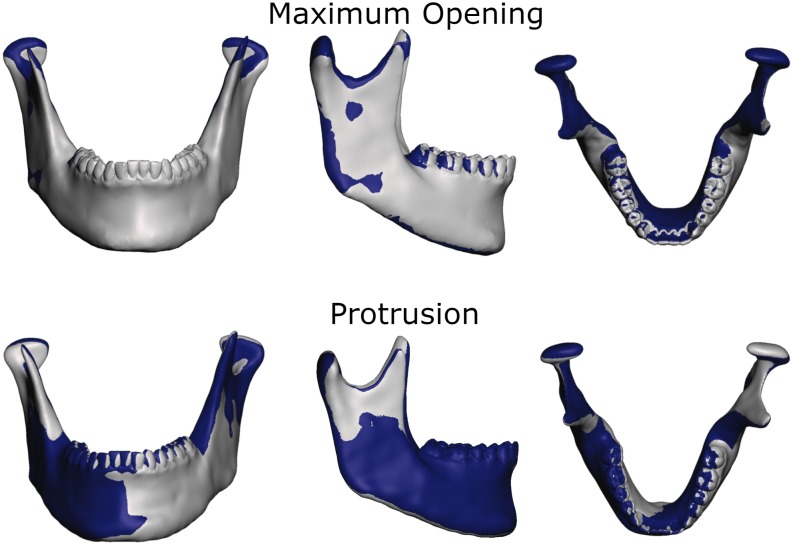
Overlay of simulation (gray) and MRI data (dark blue) for comparison of jaw position and shape for maximum opening and edge-to-edge protrusion simulations.

To more thoroughly validate the workings of our model the mandibular positions of the opening and protrusion simulation tasks were compared to high resolution meshes gathered from medical imaging data of the same volunteer. Data were collected for maximum mouth opening and a protrusive position. Figures 3, 4 show that mandible as well as disk positions for both simulation tasks fit the recorded data very well. For the disks only minor differences in shape can be observed. A detailed Hausdorff distance analysis is presented in Tables 2, 3 for opening and protrusion, respectively.

**TABLE 2:** Hausdorff distances for opening simulation.

	**Mandible**	**Discus right**	**Discus left**
Minimum distance [mm]	6^∗^10e−7	7.7^∗^10e−5	1.2^∗^10e−5
Maximum distance [mm]	1.57	3.43	2.9
Mean distance [mm]	0.42	0.84	0.61
RMS [mm]	0.53	1.1	0.88

**TABLE 3:** Hausdorff distances for protrusion simulation.

	**Mandible**	**Discus right**	**Discus left**
Minimum distance [mm]	1.1^∗^10e−6	4^∗^10e−5	1^∗^10e−6
Maximum distance [mm]	1.1	3.1	2.12
Mean distance [mm]	0.35	0.79	0.41
RMS [mm]	0.42	1.03	0.56

**FIGURE 5:**
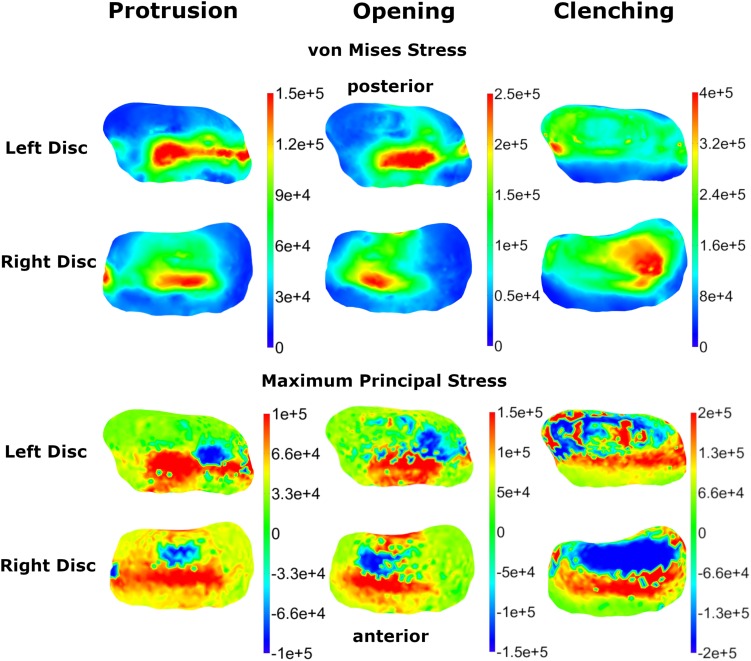
Comparison of TMJ disk von Mises and maximum principal stresses for three simulation tasks (colormaps in Pascal).

**FIGURE 6:**
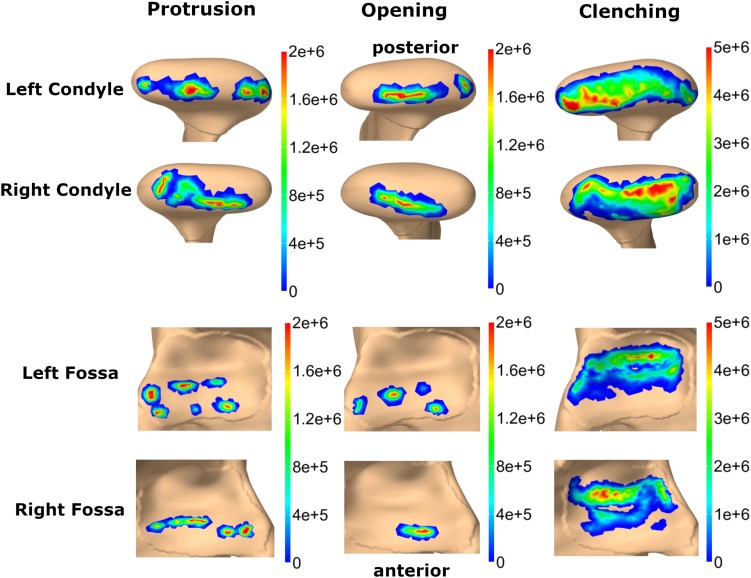
Comparison of contact pressure maps for fossa articularis and mandibular condyle for three simulation tasks (colormaps in Pascal).

Von Mises and maximum principal stresses on the disk and contact pressures on the articular cartilage are plotted in Figures 5, 6 for opening, protrusion and clenching tasks. The results show that the maximum disk stresses during opening occur in the central joint area, with a computed maximum von Mises value of around 250 kPa and a maximum principal stress of 150 kPa. For protrusion, some stress can be seen on the lateral side of the disk, additional to the central area, with maximal von Mises stresses of 150 kPa and maximum principal stresses of 100 kPa. A maximum von Mises stress of 500 kPa and a maximum principal stress of 250 kPa was observed for the clenching simulation, with highest stresses over the area of the disk that is in direct contact with the articular fossa. Overall, the maximum principal stress maps show more noise than the von Mises stress maps, which is consistent with previous literature (Koolstra and van Eijden, 2005). Maximum principal stresses generally show tensile stresses in the intermediate area of the disk, which has the highest von Mises stresses. Comparing our results, we can see that contact pressures are roughly one order of magnitude higher than the maximum principal stresses in the disk. This difference of one order of magnitude has been previously reported for maximum principal stresses of FEM disk and FEM articular cartilage (Koolstra and van Eijden, 2005). The computed quality measures resulted in a mean ratio for the radius of the circumscribed sphere to the shortest edge length of 2.25 for the left disk and 1.85 for the right disk. The mean maximal dihedral angle was 113° for the left disk and 110.9° for the right disk, respectively.

**FIGURE 7:**
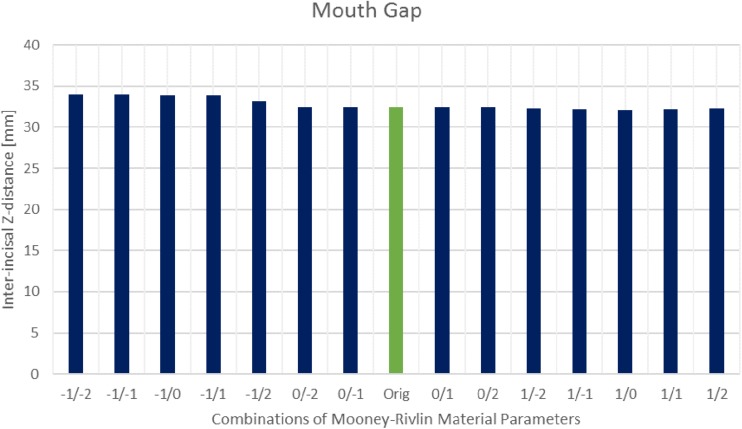
Inter-incisal distance in Z-direction for different Mooney–Rivlin material parameter combinations.

**FIGURE 8:**
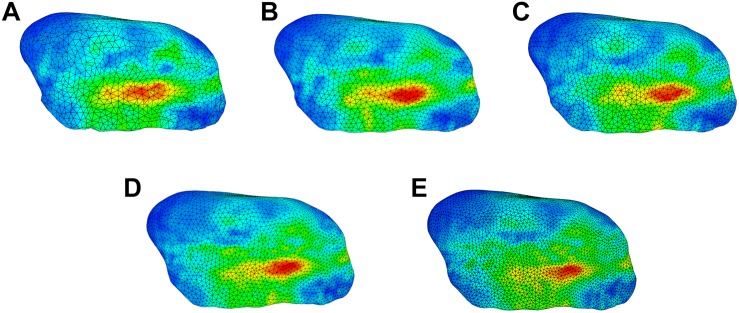
Comparison of stress trends for an opening simulation using different surface mesh edge lengths for left disk; **(A)** 0.5 mm (7522 elements per disk) **(B)** 0.45 mm (8024 elements per disk) **(C)** 0.4 mm (10167 elements per disk) **(D)** 0.35 mm (13699 elements per disk) **(E)** 0.3 mm (19175 elements per disk).

The sensitivity analysis of material properties showed that the values of the FEM parameters do not have a major influence on the mandibular end position during an opening movement (Figure 7). Only a slight shift of the mid-incisal point with a maximum of 0.24 mm laterally, 0.25 mm anterior-posteriorly and a maximum variation of 1.45 mm in z-direction was observed. A sensitivity analysis for mesh size was also conducted. Figure 8 shows the stress maps of the left disk for the end position of an opening movement using different amounts of mesh elements. The mean difference for meshes finer than 8000 elements (as used in our model) was 2.7% and the stress distributions were consistent between all mesh configurations (Figure 8).

The average execution time of a 1 s forward simulation, encompassing 1000 timesteps, was 441.03 s with a standard deviation of 1.75 s over 400 repeated simulations. Simulations were performed on a workstation PC with an Intel Xeon E5-2660 processor and 96 Gb RAM.

## Discussion

This paper presents a novel rigid body model of the complete masticatory region in combination with a detailed FEM TMJ disk and an EF representation of articular cartilage. This combination presents a novel contact formulation that has not been presented previously. The model allows for the simulation of complex movement tasks as well as high force tasks, like clenching and grinding, while achieving reasonable simulation times. This combination of complexity and computational performance helps to bring biomechanical simulations closer to clinical relevancy and enables the use of large-scale sensitivity analysis for the investigation of uncertainty in predictions of modeling parameters.

To validate our model, we compared the results of basic simulation tasks to literature values as well as high-resolution image data. The recorded inter-incisal separation for passive opening was 5 mm, which is consistent with literature values of 3 ± 2 (Langenbach and Hannam, 1999; Stavness et al., 2006). Moreover, the Hausdorff distance between the simulated mandible maximum opening position and the recorded position from MRI was extremely close, with a mean Hausdorff distance of 0.42 mm. Similar accuracy can be seen for TMJ disk positions. For maximum opening, we used a forward simulation that fully activated opening muscles and held the maximal position for 0.25 s to let the disk deform, since the position was also held for some time during MRI acquisition. For protrusion, the MRI data does not represent a maximum protrusion posture, because this position caused discomfort for the volunteer. Instead a protrusive movement until the upper and lower incisors were edge-to-edge was performed. Activation of the lateral pterygoid muscles alone was not able to protrude the mandible and hold it in this submaximal position, hence we added low level activation of the jaw closing and opening muscles to stabilize the mandible. The muscle activation levels were found by using a forward-dynamics tracking simulation that moved and held the mandible at the desired protrusive position. The resulting simulations show that our model performs well and that the computed positions for both disks and the mandible are close to the actual data for the opening and protrusion tasks.

While the computed maximum inter-incisal gap of 30 mm agrees with previous simulation studies (Peck et al., 2000; Koolstra and van Eijden, 2005; Stavness et al., 2006), it is not in the range reported for *in vivo* studies. Wide mouth opening *in vivo* is facilitated by a translation of the hyoid together with backwards rotation of the head (Muto and Kanazawa, 1994). By translating the hyoid bone in our model caudally and posteriorly we achieve a mouth opening that fits very well with the maximum opening of the volunteer recorded in the MRI in a lying position without head rotation. Hence, it is a reasonable assumption that the computed maximum mouth opening is correct and for a larger mouth gap a head rotation would have to be added.

Two major challenges of biomechanical computer investigations are the limited amount of information on properties of human tissues and the large amount of required computational power. In our opinion, one way of tackling these goals is by using appropriately complex representations for the level of information on various anatomical structures. In the case of the presented model we use a full FEM approach for the TMJ disk, which is a well-studied tissue with many investigations on its structure, composition and mechanical properties (Detamore and Athanasiou, 2003; Tanaka et al., 2008; Stankovic et al., 2013). On the other hand, TMJ articular cartilage is not as well-defined and hence we decided to speed up simulation by using a simplified elastic contact foundation approach, which has proven to be a valuable tool for modeling of cartilage layer attached to a bone (Blankevoort et al., 1991; Bei and Fregly, 2004). By using this combination of FEM and EF contact, an approach that to the best of our knowledge has not been presented previously, we can compute realistic deformation patterns for the disk and cartilage layers, while achieving simulation times much shorter than a full FEM simulation. Of course, if future research will more clearly define mechanical properties of other involved structures and a specific research question requires this step, our model and software toolkit are capable of using a full FEM approach for all parts of the model, albeit at a higher computational cost.

One limitation of the current iteration of the model is the use of a Mooney–Rivlin material for the TMJ disk. Previous investigations have shown that the disk demonstrates hyperelastic as well as viscoelastic properties and a variety of material models of different complexity have been suggested (Koolstra et al., 2007; Commisso et al., 2015, 2016). The Mooney–Rivlin material was used in the previous combined rigid body – FEM jaw model (Koolstra and van Eijden, 2005) and hence we used it for model testing. The sensitivity analysis of the material parameters also showed that the model computes quite similar mouth openings for a large range of material properties, which suggests that the dynamic behavior of the mandible is not extremely sensitive to the material properties of the TMJ disk. Moreover, the sensitivity analysis showed that larger changes of the C1 material constant of the Mooney–Rivlin material have a bigger influence on the stability of the simulations. Extremely stiff C1 (two orders of magnitudes higher) and extremely soft C1 (two orders of magnitudes lower) tend to create unstable simulations, while changes of two orders of magnitude for C2 still lead to stable simulations with only small differences in mandible position. To further improve the investigation of joint loads and disk deformation we nevertheless plan on implementing a more complex, poroelastic material model in the future.

To further verify our simulation results we performed a mesh independent grid test. The use of a complex contact model in combination with rigid body- FEM attachments makes a traditional mesh convergence study difficult to perform. For example, the number and location of attachment vertices changes for different mesh set-ups (and consequently different node locations), which inherently leads to differences in attachment between the meshes. Also, the contact force is distributed over all interpenetrating vertices, which again changes between meshes. The complexity of this problem is supported by the fact that previous models of the human masticatory system did not include a mesh independent grid analysis (Koolstra and van Eijden, 2005; Mori et al., 2010; Commisso et al., 2014, 2015; Hattori-Hara et al., 2014; Martinez Choy et al., 2017). Additionally, the purpose of the presented model and the large amount of uncertainty in measured parameters of the jaw region will permit a larger amount of error. For example, material properties of the components are largely based off of animal studies (Tanaka et al., 2002; Koolstra et al., 2007; Singh and Detamore, 2008) and the use of patient specific geometry infers a non-negligible amount of variation as well. For these reasons, the stress values computed by the model should only be used for comparative investigations (Tsouknidas et al., 2013).

In this study, we report von Mises and maximum principal stresses for three simulations tasks. In agreement with the point made in the previous paragraph, clenching stress values in literature change in the order of 1–2 magnitudes for different material models and simulation set-ups, which makes a direct comparison of results difficult. Generally, our computed trends agree with previous studies, as described in Section “Results”. As expected, clenching created high von Mises stresses over the whole disk surface which is in contact with the articular surfaces. The highest stresses were located around the latero-posterior region, consistent with the overall muscle force direction of the closing muscles. For opening and protrusion the presented von Mises stress patterns are reasonable in the context of the disk movements, which were validated using MRI data of the volunteer. Conceptually, von Mises stresses are strongly related to tissue strain and are hardly comparable to contact pressures. Articular cartilage contact pressures in our simulation are computed as pressure in the normal direction of the surface and are hence closer related to principal stresses of the disk.

The presented dynamic model highlights some unique possibilities for future investigations. For example, detailed investigations of joint loads for muscle driven mastication cycles are possible, as well as studies on the effect of TMDs, like disk dislocation, on tasks like chewing or speaking. Another problem of interest is the investigation of tooth grinding and its possible connection to increased TMJ loads. We previously presented an optimization approach that enables the use of movement as well as constraint reaction forces for forward-dynamics tracking simulations (Sagl et al., 2019a). The combination of this optimization approach with the presented model allows for a detailed investigation of muscle activation patterns and the joint loads during dynamic tooth grinding tasks. Additionally, computational modeling of the masticatory system has proven to be a useful instrument for many orofacial applications outside of the more traditional fields of dentistry. Possibilities include the use for the investigation novel TMJ total joint replacement devices (Detamore et al., 2007; Ackland et al., 2015, 2018; Omidi et al., 2018) as well as areas of orofacial function like speech (Anderson et al., 2015; M. Harandi et al., 2017) and swallowing (Mayer et al., 2016; Wang et al., 2018).

## Conclusion

This paper presents a novel computer model of the masticatory region that combines rigid body bones and Hill-type muscles with a detailed representation of the TMJ. The model allows for the investigation of dynamic tasks of the jaw apparatus, while enabling detailed investigation of stresses on the cartilage layers of the joint. The unique combination of a FEM disk and two EF articular cartilage layers allows us to keep simulation times reasonable, which is of utmost importance for the translation of computational biomechanics to the clinical practice. This will potentially lead to the development of therapeutic interventions for the restoration of orofacial functions and an increase in quality of life for TMD patients.

## Data Availability

The datasets generated for this study are available on request to the corresponding author.

## Ethics Statement

The studies involving human participants were reviewed and approved by the Institutional Review Board of the Medical University of Vienna. The patients/participants provided their written informed consent to participate in this study.

## Author Contributions

BS, MS-S, EP, MK, and IS conceived and designed the study, and analyzed and interpreted the data. BS and IS developed the computer model. BS drafted the manuscript. All authors edited the manuscript.

## Conflict of Interest Statement

The authors declare that the research was conducted in the absence of any commercial or financial relationships that could be construed as a potential conflict of interest.

## Footnotes

1 www.artisynth.org
